# A Patient-Oriented Implementation Strategy for a Perioperative mHealth Intervention: Feasibility Cohort Study

**DOI:** 10.2196/58878

**Published:** 2025-01-14

**Authors:** Daan Toben, Astrid de Wind, Eva van der Meij, Judith A F Huirne, Johannes R Anema

**Affiliations:** 1 Department of Public and Occupational Health Amsterdam UMC location Vrije Universiteit Amsterdam Amsterdam The Netherlands; 2 Societal Participation & Health Amsterdam Public Health research institute Amsterdam The Netherlands; 3 Department of Public and Occupational Health Amsterdam UMC location University of Amsterdam Amsterdam The Netherlands; 4 Department of Obstetrics and Gynaecology Amsterdam UMC location University of Amsterdam Amsterdam The Netherlands

**Keywords:** perioperative care, recovery, feasibility, convalescence, patient-oriented, surgery, perioperative, eHealth, mHealth, tailor, customize, patient care, digital intervention, health intervention, patient education, surgical care, hospital care, digital health, perioperative medicine, elective surgery, technology, caregiver, mobile app, digital care

## Abstract

**Background:**

Day surgery is being increasingly implemented across Europe, driven in part by capacity problems. Patients recovering at home could benefit from tools tailored to their new care setting to effectively manage their convalescence. The mHealth application ikHerstel is one such tool, but although it administers its functions in the home, its implementation hinges on health care professionals within the hospital.

**Objective:**

We conducted a feasibility study of an additional patient-oriented implementation strategy for ikHerstel. This strategy aimed to empower patients to access and use ikHerstel independently, in contrast to implementation as usual, which hinges on the health care professional acting as gatekeeper. Our research question was “How well are patients able to use ikHerstel independently of their health care professional?”

**Methods:**

We investigated the implementation strategy in terms of its recruitment, reach, dose delivered, dose received, and fidelity. Patients with a recent or prospective elective surgery were recruited using a wide array of materials to simulate patient-oriented dissemination of ikHerstel. Data were collected through web-based surveys. Descriptive analysis and open coding were used to analyze the data.

**Results:**

Recruitment yielded 213 registrations, with 55 patients ultimately included in the study. The sample was characterized by patients undergoing abdominal surgery, with high literacy and above average digital health literacy, and included an overrepresentation of women (48/55, 87%). The implementation strategy had a reach of 81% (63/78), with 87% (55/67) of patients creating a recovery plan. Patients were satisfied with their independent use of ikHerstel, rating it an average 7.0 (SD 1.9) of 10, and 54% (29/54) of patients explicitly reported no difficulties in using it. A major concern of the implementation strategy was conflicts in recommendations between ikHerstel and the health care professionals, as well as the resulting feelings of insecurity experienced by patients.

**Conclusions:**

In this small feasibility study, most patients were satisfied with the patient-oriented implementation strategy. However, the lack of involvement of health care professionals due to the strategy contributed to patient concerns regarding conflicting recommendations between ikHerstel and health care professionals.

## Introduction

Day surgery—defined as admittance to and discharge from a hospital within 24 hours following surgery—has seen a marked increase in Organisation for Economic Co-operation and Development member countries over the past decades [1]. The appeal of day surgery derives from multiple factors, including its reduced cost, decreased morbidity and mortality, and high levels of patient satisfaction [2-6]. When it comes to postsurgical recovery, however, the reports are more nuanced. Tran et al [7] showed how 1 in 3 patients exhibit suboptimal recovery trajectories following day surgery. Patients recovering at home describe feelings of insecurity, an experience moderated by the timely provision of information, professional support, and expectation management [4,8-12]. mHealth interventions have been shown to be effective when it comes to targeting these domains and their use in the perioperative setting is well appreciated by patients [13,14]. In the Netherlands specifically, the Patient Journey app has been shown to improve postoperative outcomes for patients with musculoskeletal disorders [15].

Similarly, the mHealth intervention ikHerstel (meaning “I recover” in Dutch) is a tool designed to support patients undergoing abdominal surgery during their perioperative period. The intervention’s ability to speed up postoperative recovery, reduce pain, and improve patients’ quality of life has been established in previous studies [12,16-18]. However, its implementation occurs on the level of the hospital ward, and it hinges on the involvement of health care professionals within the ward, who act as both distributors of the intervention and instructors of patients. This strategy features benefits as well as challenges: health care professionals are well situated to select eligible patients and can improve adherence to treatment when they use effective communication strategies [19,20]. However, at the time of publishing, the intervention has been implemented in only 10% of hospitals in the Netherlands. Wider implementation is hampered by, among other factors, financial barriers present in the Dutch health care system that make upscaling of telemonitoring interventions in general a difficult enterprise [21]. This limits ikHerstel’s reach, leaving patients bereft of its aforementioned benefits.

In this feasibility study, we explored a patient-oriented implementation strategy for ikHerstel that aimed to circumvent this hospital-level barrier by targeting patients directly. If successful, this strategy could operate in addition to implementation as usual, with reimbursement flowing from health insurers to patients. We therefore aimed to evaluate whether it would be successful in increasing the intervention’s reach and whether patients, once reached, were able to use ikHerstel independently from their health care professional.

## Methods

### Ethical Considerations

Approval for the study was granted by the medical ethics committee of Amsterdam University Medical Center on May 31, 2022 (2022.0224). Informed consent was obtained through postal mail and patients were informed of their ability to opt out of participation in the study at any time. Patients were provided with access to ikHerstel free of charge but were not offered any remuneration for their participation in the study. Data were deidentified by the coordinating researcher, and patients were labeled using random strings. The patient identification keys were kept in a separate location from the data.

### Study Setting

We conducted a prospective study assessing the feasibility of a patient-oriented implementation strategy for the ikHerstel mHealth intervention. Our assessment was performed based on the model of Steckler and Linnan [22]; its outcomes were reach, dose delivered, dose received, fidelity, and recruitment. In consultation with health insurers and a patient interest group, we aimed to include 100 perioperative patients representing the theoretical user base of the ikHerstel app, that is, any patients who were theoretically able to access the app and use it in such a way as to manage their own recovery, regardless of age, gender, nationality, literacy, digital literacy, or health literacy. Recruitment started in September 2022 and lasted through September 2023.

### Inclusion and Exclusion Criteria

Patients were eligible for inclusion if they were older than 18 years, proficient in the Dutch language, and prospective recipients of one of the following elective surgical procedures: laparoscopic or abdominal hysterectomy, laparoscopic cholecystectomy, open or laparoscopic inguinal hernia surgery, or laparoscopic adnexal surgery. Patients were excluded if the date of their surgery was ≥14 days prior to inclusion, they were undergoing a combination of surgeries, they had comorbidities that invalidated the convalescence recommendations provided by ikHerstel, they were undergoing oncological surgery, or they were receiving care from a hospital that had already implemented ikHerstel.

### Intervention and Procedure

ikHerstel was developed in collaboration with health care professionals of a diverse background. Its development process has been described previously [23]. An overview of the current functions and layout of ikHerstel is provided in Multimedia Appendix 1. Its aim is to prepare patients and manage their expectations preoperatively and to support them in recovery of the daily functions of life postoperatively [23]. Each patient received the ikHerstel intervention in addition to usual care. Patients were able to interact with the intervention in the form of a mobile app, which they used up to the point of their total recovery. They were provided with personal accounts in which they constructed their recovery plan through goal attainment by selecting 8 personal activities from a list of 31 to constitute their most important recovery goals. In this way, one patient might create a plan focused on performing tasks around the house while another might create one centered on regaining the ability to run long distances. Patients monitored their recovery plan through the mobile app: they were asked to indicate when they were able to perform each of the activities in their plan. The total postoperative recovery was visible as a percentage within the app. Additionally, educational material about recovery was provided to patients in the form of text and video animations through the app’s library screen.

### Implementation Strategy as Usual

In its current form, ikHerstel’s implementation strategy hinges on health care professionals, who recruit eligible patients, introduce them to the app and its potential benefits, and provide them with access by creating a personal account. This final step is particularly crucial, as patients cannot access ikHerstel without an account, and health care professionals preload each account with recovery-related data specific to the patient’s surgical procedure. Implementation occurs at the level of the hospital ward. A medical liaison associated with ikHerstel trains the ward’s staff in the app’s use and goals and in carrying out support tasks like creating patient accounts. The hospital ward is also provided with a web portal that mediates these administrative functions, allows for monitoring of each patient’s recovery, and provides health care professionals with organizational support.

### Patient-Oriented Implementation Strategy

**Figure 1:**
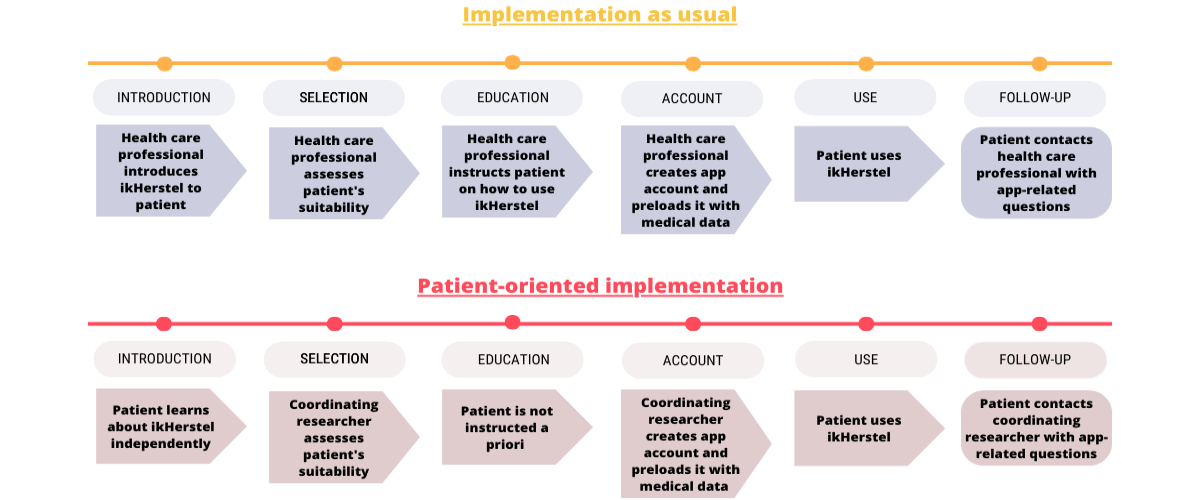
Schematic representation of the differences between implementation as usual and the patient-oriented implementation.

The patient-oriented implementation strategy piloted in this study circumvented health care professionals, relying instead on patients to sign up and use ikHerstel independently. Health care professionals did not have access to the app or the web portal. Instead, these responsibilities were assigned to the coordinating researcher as a placeholder for the support staff of the ikHerstel spinoff company. During the course of the study, the coordinating researcher created patients’ accounts and loaded them with surgery-related data based on information provided by the patients. Patient monitoring through the web portal was not performed. In case of questions concerning ikHerstel, patients were directed to the coordinating researcher, whose contact details were provided. Patients with medical questions were directed by the researcher to consult their health care professional. This highlights the key role still reserved for health care professionals in this patient-oriented implementation, as they retained responsibility for care of their patients, including monitoring for adverse outcomes. Accordingly, patients were informed that their health care professional held final authority over the content and provision of care. Figure 1 illustrates the differences between the implementation strategies. Table 1 presents an overview of the recruitment tools that were used, distinguishing between hospital-independent and -dependent tools.

With the exception of the magazine advertisements, all advertisements followed the same basic design, created with low-literacy patients in mind. An example is provided in Multimedia Appendix 2. These materials were distributed to patients in hospitals, on patient fora, on webpages of patient interest groups, in patient magazines, through internet search engine advertisements, and within patient groups on social media. Each advertisement linked to a web portal where patients were informed of the study and asked to leave their contact details. Patients were subsequently contacted via telephone by the coordinating researcher, who provided further information and performed screening on the basis of the inclusion and exclusion criteria.

**Table 1:** Materials used for study recruitment and the frequency of their use, split into hospital-dependent and -independent tools.

Materials		Frequency of use, n
**Hospital-independent**		
	Forum advertisements	15
	Webpage advertisements	2
	Internet search engine advertisements	1
	Social media advertisements	4
	Magazine advertisements	2
**Hospital-dependent**		
	Flyers	11
	Posters	10
	Business cards	6
	Electronic displays	5
	Hospital staff	2

### Data Collection

Data were collected through a set of 4 digital surveys constructed, distributed, and maintained through Survalyzer (Survalyzer AG). A baseline survey (T_0_) was used to collect demographic data. Follow-up surveys were distributed to patients at T_1_ (3 weeks), T_2_ (6 weeks), and T_3_ (12 weeks) after surgery to collect data on the user experience.

### Background Factors and Implementation Outcomes

Demographic data included socioeconomic factors like age, sex, and education level, which is aligned with a previous study by van der Meij et al [24]. Demographics also included a measure of patients’ traditional literacy, operationalized on the basis of the Diagnostic Illiteracy Scale, where a score of 14 points or higher constitutes a risk of the individual being illiterate [25]. Digital literacy was operationalized using patient self-assessment and a scanning tool (Quickscan) developed for physicians by the Dutch patient advocate organization Pharos, which characterizes patients as digitally unskilled with a score of 10 points or higher [26].

The model by Steckler and Linnan [22], commonly used in public health, describes the evaluation of implementation outcomes as a concatenated appraisal of an intervention’s context, reach, dose delivered, dose received, fidelity, and recruitment. Operationalization of these outcomes was performed similarly to previous process evaluations of ikHerstel to facilitate comparison [24,27]. We omitted the aspect of fidelity, as the app does not deviate from protocol in its delivery of the intervention. We also omitted context, as this is described in earlier publications, as well as the aspect of implementation, as we judged its transformation of the other aspects into a summative score to be a bad fit for our study. We also evaluated the recruitment tools and their channels (hospital dependent vs independent) in terms of their effectiveness in recruiting eligible patients to use the app. To compute this count, we asked patients to state how they were informed about the study.

We measured patient attitudes in alignment with the patient-oriented character of the implementation strategy and for comparison with previous research [24,27]. We operationalized patient attitudes as patients’ self-reported satisfaction rating and their experienced barriers to use. We additionally measured patient attitudes using the unified theory of acceptance and use of technology 2 (UTAUT2), developed by Venkatesh et al [28]. Briefly, this framework describes an individual’s intention to use a technology as being determined by 7 constructs: performance expectancy, effort expectancy, social influence, facilitating conditions, hedonic motivation, price value, and habit. Social influence and hedonic motivation were deemed less relevant to ikHerstel’s context and thus were not included. Relevant UTAUT2 survey items were selected by the researchers, adapted to the research context, and translated into Dutch. Response categories followed a 4-point Likert scale centered on agreement. The resultant survey is provided in Multimedia Appendix 3. A full overview of the study’s outcomes and their operationalization is presented in Table 2.

**Table 2:** Operationalization of implementation outcomes and patient attitudes.

		Description	Operationalization
**Implementation outcomes^a^**			
	Reach	The proportion of the intended target audience that participated in the study	Numerator: number of patients who met the inclusion criteria and signed an informed consent form; denominator: number of patients who met the inclusion criteria, regardless of their eventual participation in the study
	Dose delivered	The number or amount of intended units of the intervention provided to the study population	Numerator: number of patients who were provided with an account for the ikHerstel app; denominator: number of patients who met the inclusion criteria and signed an informed consent form
	Dose received	The extent to which participants actively engaged with, interacted with, were receptive to, or used the intervention	Numerator: number of patients who activated their ikHerstel account, created a recovery plan, and used the app on a weekly basis; denominator: number of patients who were provided with an account for the ikHerstel app
	Recruitment	The effectiveness of the procedures used to attract participants	An appraisal of the effectiveness of each recruitment medium (hospital dependent vs independent) and tool in terms of the number of inclusions versus registrations they produced
**Patient attitudes**			
	Patient satisfaction	—^b^	Patient satisfaction, assessed through a self-reported score between 0 and 10
	Barriers to use	—	Five open questions: What did you like about using ikHerstel? What makes using ikHerstel easy? What did you dislike about using ikHerstel? What makes using ikHerstel difficult? Do you have any other comments about the ikHerstel app?
	Performance expectancy^c^	The degree to which using the technology will provide benefits to consumers	The degree to which patients view ikHerstel as being able to beneficially affect their postsurgical recovery; operationalized as 3 self-reported items, scored using a 1-4 Likert scale
	Effort expectancy^c^	The degree of ease associated with consumers’ use of the technology	The degree to which patients feel using ikHerstel is simple and straightforward; operationalized as 3 self-reported items, scored using a 1-4 Likert scale
	Facilitating conditions^c^	Consumers’ perceptions of the resources and support available to perform a behavior	The degree to which patients feel they are supported in their use of ikHerstel; operationalized as 2 self-reported items, scored using a 1-4 Likert scale
	Price value^c^	Consumers’ cognitive tradeoff between the perceived benefits of the technology and the monetary cost for using it	The degree to which patients are willing to pay for their use of ikHerstel; operationalized as 1 self-reported item, scored using a 1-4 Likert scale
	Habit^c^	The extent to which consumers tend to perform behaviors automatically because of learning	The degree to which patients feel their use of ikHerstel has become habitual; operationalized as 1 self-reported item, scored using a 1-4 Likert scale

^a^Based on the model by Steckler and Linnan [22].

^b^Not applicable.

^c^Based on the unified theory of acceptance and use of technology 2 by Venkatesh et al [28].

### Data Analysis

Descriptive statistics were used to summarize the study’s findings according to each process outcome as well as the UTAUT2 dimensions. Open-ended patient attitude items were assessed and categorized by the coordinating researcher, and the resultant categories were subsequently reviewed by another researcher from the research team.

## Results

### Reach

**Figure 2:**
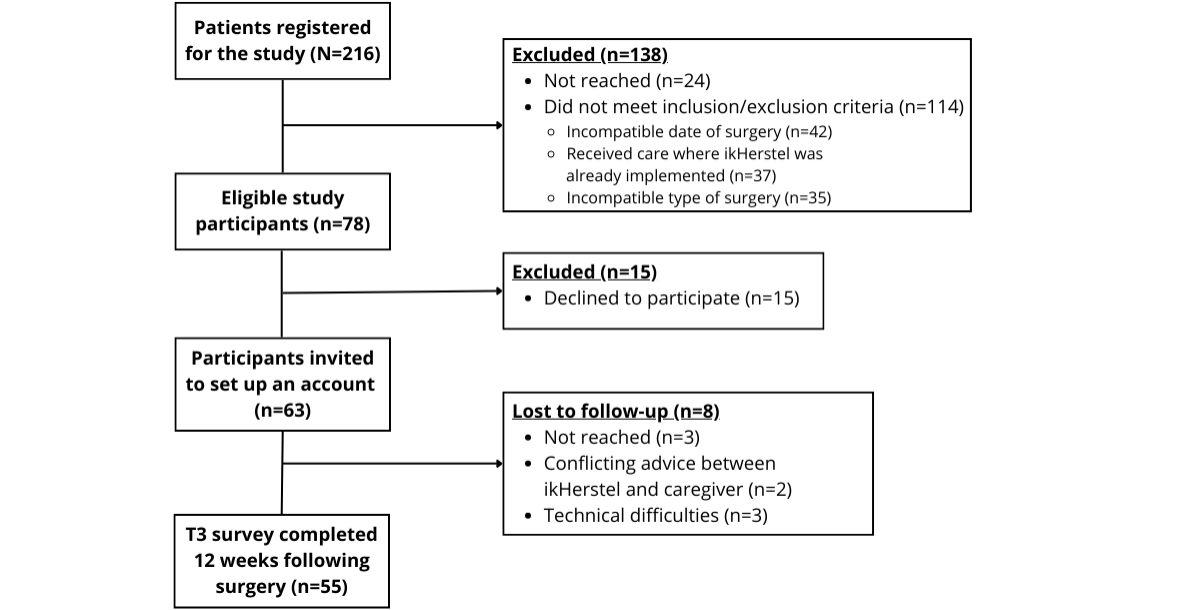
Flow chart for inclusion in the study.

In the period between September 2022 and September 2023, 216 patients registered for the study. A schematic representation of the inclusion process is presented in Figure 2. Initial screening via telephone resulted in 148 exclusions. A major reason for exclusion was timing, as many patients only signed up for ikHerstel once their surgery had already taken place. The exclusion criteria were revised to account for this unexpected result, allowing patients to participate up to 14 days following their surgery. This nevertheless still led to 42 exclusions due to timing. A total of 68 patients were identified as eligible for participation and were subsequently sent informed consent forms. Among these 42 patients, 5 were excluded due to incompatible types of surgery that had not been identified as such prior to telephone screening. This resulted in a total of 63 included patients, which constitutes a reach of 81% (63 / (216 – (109 + 5 + 24)).

Baseline characteristics of these respondents are presented in Table 3. A majority of respondents were female, corresponding to one half of the included surgery types being gender specific for women. All the respondents had Dutch nationality and close to two-thirds (35/55) had a high level of education. All patients scored full points on the Quickscan test, and only one respondent gave a categorical self-description as being not very digitally skilled. The same held true for traditional literacy, with none of the respondents scoring in a range that would put them at risk of having low literacy skills [29].

**Table 3:** Sample characteristics (n=55).

Variables			Values
**Age (years), mean (SD)**			48.6 (12.4)
**Sex, n (%)**			
	Male	7 (13)	
	Female	48 (87)	
**Nationality, n (%)**			
	Dutch	55 (100)	
**Education, n (%)**			
	Low	7 (13)	
	Intermediate	13 (24)	
	High	35 (64)	
**Type of surgery, n (%)**			
	Laparoscopic uterus extirpation	21 (38)	
	Abdominal uterus extirpation	8 (15)	
	Vaginal uterus extirpation	6 (11)	
	Laparoscopic adnexal surgery	5 (9)	
	Laparoscopic cholecystectomy	10 (18)	
	Laparoscopic inguinal hernia surgery	4 (7)	
	Open inguinal hernia surgery	1 (2)	
**Digital skills—Quickscan, mean (SD)**			6 (0)
**Digital skills—self-scan (categorical), n (%)**			
	Very digitally skilled	29 (53)	
	Of average skill	25 (46)	
	Not or not very digitally skilled	1 (2)	
**Digital skills—self-scan (numeric), mean (SD)**			7.9 (1.5)
**Literacy score, mean (SD)**			8.5 (2.6)

### Dose Delivered

Of the 63 patients who signed the informed consent form and met the inclusion and exclusion criteria, 63 were provided with an account in the ikHerstel app. The dose-delivered fraction therefore computes to a percentage of 100%.

### Dose Received

Of the 63 patients who were provided with an account, 55 activated their account and created a personalized recovery plan. Of these 55 patients, 34 reported using the app on a weekly or more frequent basis. The dose received fraction (34/63) therefore computes to a percentage of 54%.

### Recruitment

An overview of the number of registrations and inclusions per recruitment tool is provided in Table 4. Most of the registrations (87/216, 40%) originated from tools that were dependent on hospitals, like posters, waiting room electronic displays, and hospital staff. Tools outside of the hospital yielded 36% (77/216) of registrations. However, they yielded more eligible patients (32 vs 31), as well as a higher proportion of eligible patients (32/77) compared to hospital-dependent tools (31/87).

**Table 4:** Overview of the number of registrations and eligible patients per recruitment tool.

Tools		Registrations, n (N=216)		Eligible patients, n (n=63)
**Hospital-independent**				
	Forum advertisements		14	8
	Webpage advertisements		1	0
	Internet search		17	7
	Social media		18	13
	Magazine advertisements		21	2
	Other^a^		6	2
	Subtotal		77	32
**Hospital-dependent**				
	Flyers		11	6
	Posters		10	6
	Business cards		2	2
	Electronic displays		11	7
	Hospital staff		24	8
	Unspecified^b^		27	0
	Other^c^		2	2
	Subtotal		87	31
	Unknown^d^		52	0

^a^This category included person-to-person contacts (n=5) and receiving an email of unknown origin (n=1).

^b^These respondents stated that the hospital was the source of their contact with ikHerstel.

^c^This category included patient-to-patient contacts in the convalescence room (n=1) and the webpage of the hospital (n=1).

^d^These respondents did not state how they came into contact with ikHerstel, mostly due to a lack of communication or stated interest on their part.

### Patient Attitudes

Patients rated their overall satisfaction with ikHerstel an average 7.0 (SD 1.9) of 10. One patient did not answer the open-ended questions. A substantial proportion of patients (14/54) explicitly stated not having any dislikes about using ikHerstel, and an even greater proportion (29/54) explicitly reported no difficulties in using it. Most patients (49/54) reported positive experiences with ikHerstel. The most frequently stated (17/49) positive experience with ikHerstel related to its provision of perspective when it came to recovery. Patients furthermore found the app was clear in its presentation of information (10/49) and easy to use (8/46). Other stated likes related to the app’s motivating power (6/49), its function as a source of information (3/49), its comforting effect (2/49), the patients’ ability to benchmark their recovery (2/49), and a general statement of satisfaction (1/49). A majority of patients (50/54) reported on aspects that made using ikHerstel easy. The most frequently stated aspect was its clarity in presenting information (23/50). Patients also found it easy to navigate through the app (20/50) and praised its round-the-clock availability as a mobile phone app (6/50). One patient simply affirmed its ease of use, and others (4/50) found nothing about it easy. One patient stated, “Easy to use and provides motivation to start exercising and pick up activities again.”

The most striking dislikes reported by patients were those concerning its recommendations. In some cases, what the app prescribed was misaligned with what patients felt they could handle. This mismatch ran both ways, as some patients felt the app was too ambitious, while others reported it was holding them back: “..that you [ikHerstel] go much faster than my recovery. That feels like failure because it repeatedly says you are behind on your recovery. It became more and more frustrating.”

Another frequently stated mismatch was between ikHerstel and health care professionals. Of the 45 patients who reported receiving recovery recommendations from their health care professional, 33 stated that the recommendations provided by ikHerstel conflicted “sometimes” or more frequently. The majority of these (n=17) described the health care professional as conservative when it came to performing activities compared to the app. Others (n=8) reported that the app’s recommendations were more elaborate and covered a wider slice of their daily life. Some patients (n=6) explicitly stated a dislike of the mismatch. In these cases as well, health care professionals’ prescriptions were more conservative, and as a result, these patients reported feelings of frustration and insecurity: “[T]he recommendations from both the hospital and the GP [general practitioner]’s assistant were so much more conservative regarding when you should try and pick up activities that it made me feel insecure.”

Other dislikes related to difficulties with inputting data (n=14), a lack of personalization (n=7), a lack of functionalities (n=5), the demotivating effect of the app (n=3), accessibility (n=1), technical failures (n=1), and miscellaneous difficulties (n=3); 14 patients found nothing to dislike. One patient stated, “After altering one of the activities, I had to redo all the input I had previously provided.”

### UTAUT2 Dimensions

Among UTAUT2 survey dimensions, respondents rated their performance expectancy an average of 2.7 (SD 0.8) of 4 points. Effort expectancy was rated at 3.3 (SD 0.8) of 4 points and facilitating conditions at 3.4 (SD 0.7) of 4 points. The dimension of price value was scored an average 1.7 (SD 0.7) of 4 points, corresponding to 6 of 55 patients confirming that they would be agreeable to paying for the services provided by ikHerstel. A substantial proportion of patients (20/52) stated their use of ikHerstel had become habitual, resulting in an average score of 2.3 (SD 0.9) of 4 points for the dimension of habit.

## Discussion

### Principal Findings

In this feasibility study, we aimed to evaluate a patient-oriented implementation strategy for the mHealth intervention ikHerstel. We included 55 patients undergoing abdominal surgery among 216 registrations, and we investigated whether direct distribution of ikHerstel was a feasible addition to its implementation through hospitals. Hospital-dependent recruitment yielded slightly more registrations, while hospital-independent recruitment produced more eligible patients. The patient-oriented strategy constituted a reach of 81% (63/78), and 100% of reached patients were sent the intervention, after which 54% (34/63) engaged with it. Patients reported general satisfaction with ikHerstel, scoring it an average 7.0 (SD 1.9) of 10 points.

Other studies have examined user experiences with mHealth apps in the perioperative setting. To illustrate, a cross-sectional study on the Patient Journey app yielded higher levels of satisfaction compared to this study [15]. Patients were likewise positive about the app’s ease of use and its clear provision of useful information. A systematic review of patient experiences with mHealth confirms that this is a main benefit of these interventions [13]. The finding that patients regretted losing the possibility of communicating with their health care professional through the app was not replicated in our study. A previous process evaluation concerning a version of ikHerstel that did feature this function found that patients appreciated it, but that it should not replace a telephone appointment with their health care professional [24].

We hypothesized that the patient-oriented implementation strategy would increase ikHerstel’s reach. However, in terms of absolute scale, this expectation proved incorrect. Over the span of a year, only 216 registrations were generated, compared to the 1031 and 673 reported in previous studies, where hospitals played a central role in recruitment through their waiting lists [24,27]. Despite lower registration numbers, the reach of the patient-oriented implementation strategy was better, or at least comparable to, previous studies, at 81%, compared to 40% and 60%, respectively [24,27]. In addition to scale, an advantage of recruitment through hospitals was apparent when comparing the rate of and reasons for exclusion. Only 5% of patients were excluded due to ineligibility in the study by van der Meij et al [24], compared to our study’s exclusion rate of 53%. Poor timing (n=42, 37%), double registration (n=37, 32%), and ineligible types of surgery (n=35, 31%) make up the reasons for exclusion. In fact, poor timing proved such a barrier to participation that we were forced to revise our exclusion criteria halfway through the study to include patients up to 14 days after their surgery. Our assumption that patients would start looking for tools to support them through their perioperative journey prior to surgery proved false. In practice, this means that a substantial proportion of patients missed out on ikHerstel’s preoperative functions designed to enhance preparation and manage expectations.

The mismatch between ikHerstel’s recommendations and those of health care professionals also points to the strategic position of these professionals in perioperative care. Patients listed this mismatch not only as a source of dislike but also as one of feelings of insecurity. Other studies have reported similar findings [13,15]. The conflict itself may arise due to the conservative character of many health care professionals, as some studies indicate [30,31]. Complications that arose may likewise have caused mismatches by altering patients’ needs and invalidating the care provision of ikHerstel. Both cases advocate for the integral role of health care professionals in mHealth implementation strategies, as they are ideally situated to select patients and to adjust care provision when complications arise. By replacing these agents with a researcher, we effectively placed a part of our intervention outside of the broader system of care. Despite this, most patients had no trouble using ikHerstel independently. More than half of patients reported no difficulties and a quarter of patients explicitly found nothing to dislike.

Patients find value in mHealth apps in their provision of information that would otherwise not be readily available, and find even more value if that information is tailored to the patients’ individual situation [32]. In light of our own findings, it seems vital that health care professionals are involved in how mHealth is implemented to provide this function: as gatekeepers, selecting the right patients; as anchors, integrating an intervention into the broader system of care; but not as tech support, as patients seem able to navigate mHealth independently. Health care professionals could be involved through professional training, introducing them to the mHealth evidence base, or it may take the form of colleagues operating as implementation champions [33].

### Limitations

A number of limitations need to be addressed, the first being the absence of health care professionals’ perspectives in our evaluation of the implementation strategy’s feasibility. The patient-oriented character of the study was chosen in dialogue with patient interest groups and health insurers, and aligns with the study’s aim of empowering patients to access ikHerstel even if their hospital has not implemented it. Health care professionals’ assessments of our strategy may nevertheless have yielded important insights, as they may have shed light on conflicting recovery recommendations that were received by the participants.

Another limitation is the study’s lack of a diverse sample of patients. We disproportionately included highly educated women of Dutch nationality. While an overrepresentation of women was expected due to the overrepresentation of gynecological types of surgery in our study, this does not explain the sample’s high level of education or the lack of international patients. In the case of the latter, the use of the Dutch language in our recruitment material may well have discouraged any international patients from engaging with the study. For the former, the multimedia recruitment strategy we used, emphasizing access to a medical innovation, may have selected for highly educated patients, as some studies have reported on the association between educational level and the use of health services [34-37]. Here too, we may see a reflection of the absence of a health care professional, whose prompting influence might have worked to transcend such barriers. A study on sex differences regarding intention to use mHealth apps in the Netherlands found that women had a more negative attitude of mHealth, perceiving it as being less useful than did men [38]. This may have driven the difference in overall satisfaction scores between this study and the previous study by van der Meij et al [24], who included a more equal distribution of male versus female patients. Stratification by sex provides some weight to this argument, producing an average satisfaction score of 8.3 for men versus 6.8 for women, although these figures lack reliability precisely due to our sample’s low representation of men.

### Conclusions

The patient-oriented implementation strategy evaluated in this study was an equivocal success. One of its main hypothesized advantages of more easily reaching a wide audience of patients was not demonstrated. However, its method of recruitment has low costs, and most patients were satisfied and engaged with the mHealth app. Lack of involvement of health care professionals, rather than usability issues on the patients’ side, contributed to patients’ concerns regarding conflicting recommendations between ikHerstel and health care professionals. Given patient engagement, satisfaction, and improvement in outcomes [12,16-18] with use of such apps, hospitals should consider strategies where health care professionals are involved in selecting patients that may benefit from mHealth apps for postoperative recovery after day surgery and guiding patients’ care.

## Appendix

Multimedia Appendix 1 Screening structure and content of ikHerstel.

Multimedia Appendix 2 Advertisement design.

Multimedia Appendix 3 Unified theory of acceptance and use of technology survey items.

## Abbreviations

UTAUT2: unified theory of acceptance and use of technology 2
