# Correction: A Patient-Oriented Implementation Strategy for a Perioperative mHealth Intervention: Feasibility Cohort Study

**DOI:** 10.2196/71874

**Published:** 2025-02-12

**Authors:** Daan Toben, Astrid de Wind, Eva van der Meij, Judith A F Huirne, Johannes R Anema

**Affiliations:** 1 Department of Public and Occupational Health Amsterdam UMC location Vrije Universiteit Amsterdam Amsterdam The Netherlands; 2 Societal Participation & Health Amsterdam Public Health research institute Amsterdam The Netherlands; 3 Department of Public and Occupational Health Amsterdam UMC location University of Amsterdam Amsterdam The Netherlands; 4 Department of Obstetrics and Gynaecology Amsterdam UMC location University of Amsterdam Amsterdam The Netherlands

In “A Patient-Oriented Implementation Strategy for a Perioperative mHealth Intervention: Feasibility Cohort Study” JMIR Perioper Med 2025;8:e58878) the authors noted one error.

The affiliations were incorrectly published as:

Daan Toben^1,2^, MSc; Astrid de Wind^1,2^, PhD; Eva van der Meij^2,3^, PhD; Judith A F Huirne^2,3,4^, PhD; Johannes R Anema^2^, PhD

^1^Department of Public and Occupational Health, Amsterdam UMC location Vrije Universiteit Amsterdam, Amsterdam, The Netherlands

^2^Societal Participation & Health, Amsterdam Public Health, Amsterdam, The Netherlands

^3^Department of Obstetrics and Gynaecology, Amsterdam UMC location University of Amsterdam, Amsterdam, The Netherlands

^4^Department of Public Health, Amsterdam University Medical Center, University of Amsterdam, Amsterdam, The Netherlands

These have been revised as follows:

Daan Toben^1,2^, MSc; Astrid de Wind^2,3^, PhD; Eva van der Meij^2,3^, PhD; Judith A F Huirne^2,4^, PhD; Johannes R Anema^1,2^, PhD

^1^Department of Public and Occupational Health, Amsterdam UMC location Vrije Universiteit Amsterdam, Amsterdam, The Netherlands

^2^Societal Participation & Health, Amsterdam Public Health research institute, Amsterdam, The Netherlands

^3^Department of Public and Occupational Health, Amsterdam UMC location University of Amsterdam, Amsterdam, The Netherlands

^4^Department of Obstetrics and Gynaecology, Amsterdam UMC location University of Amsterdam, Amsterdam, The Netherlands

The correction will appear in the online version of the paper on the JMIR Publications website on February 12, 2025 together with the publication of this correction notice. Because this was made after submission to PubMed, PubMed Central, and other full-text repositories, the corrected article has also been resubmitted to those repositories.

