# Supplementary figures and images for: A Patient-Oriented Implementation Strategy for a Perioperative mHealth Intervention: Feasibility Cohort Study
Source: JMIR Perioper Med. 2025 Jan 14;8:e58878. doi: 10.2196/58878 (PMC11775485; doi:10.2196/58878)

## Appendix A:

## Screen structure and content of ikHerstel

**
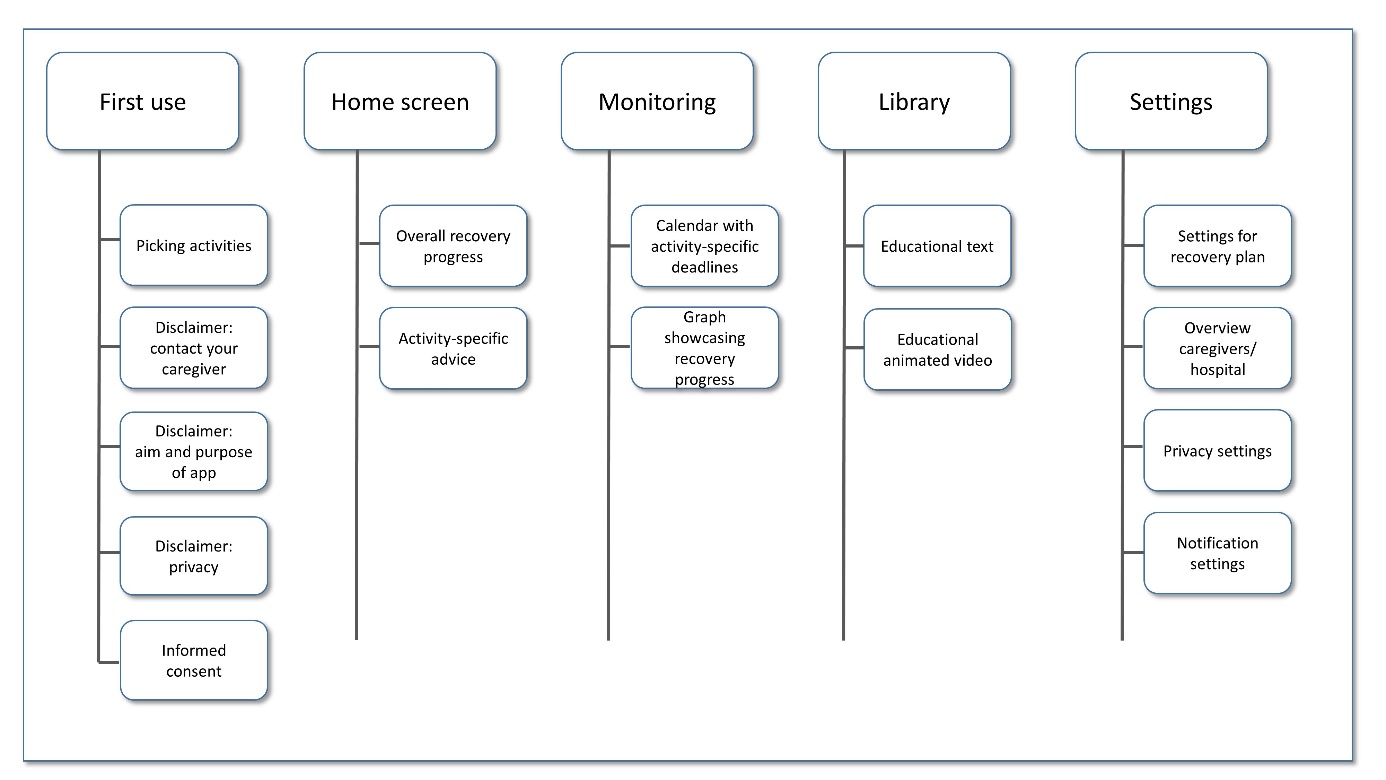
**

## Screenshots of ikHerstel

**
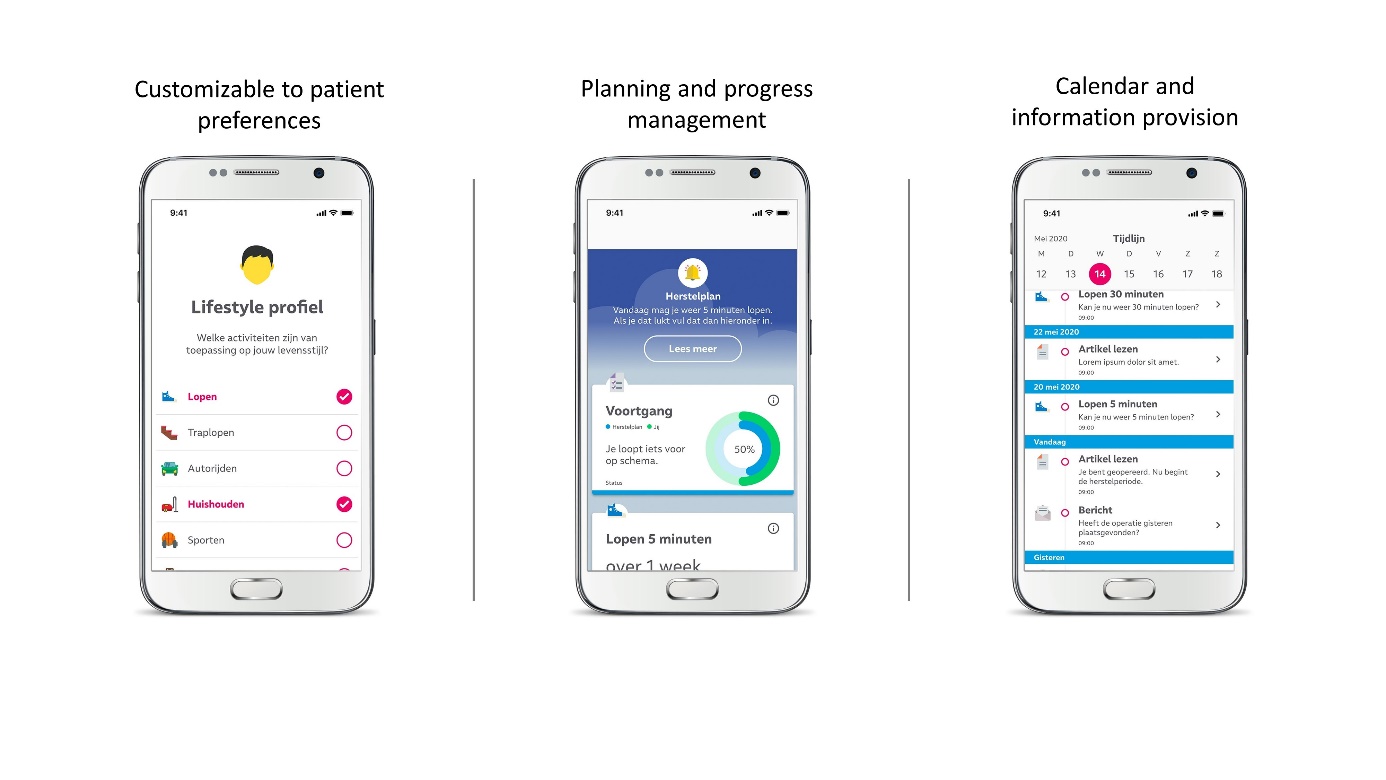
**

Supplement: Multimedia Appendix 1 [file periop_v8i1e58878_app1.docx]

## Appendix B: Ad design


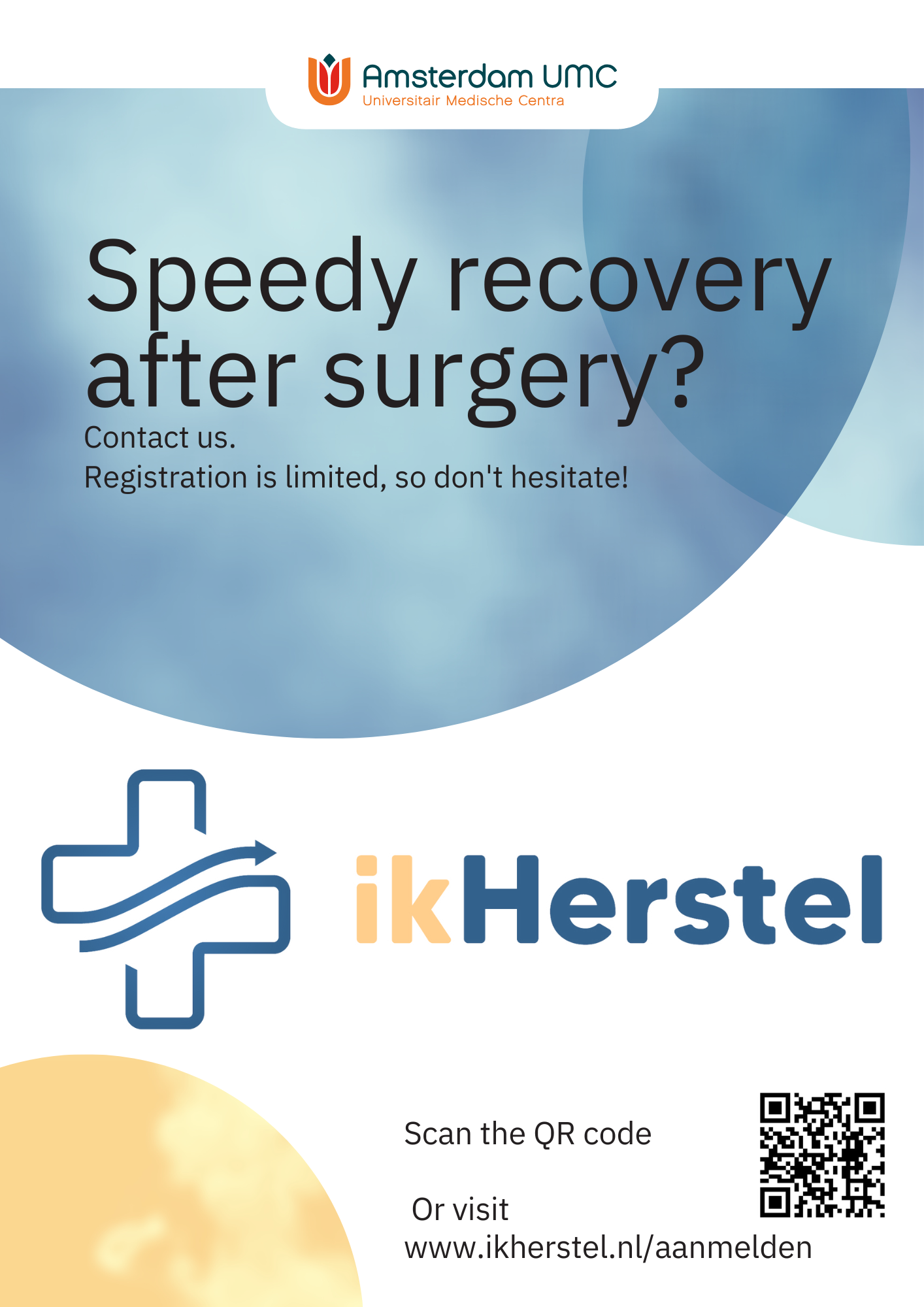

Supplement: Multimedia Appendix 2 [file periop_v8i1e58878_app2.docx]
