# Supplementary material for: A Patient-Oriented Implementation Strategy for a Perioperative mHealth Intervention: Feasibility Cohort Study
Source: JMIR Perioper Med. 2025 Jan 14;8:e58878. doi: 10.2196/58878 (PMC11775485; doi:10.2196/58878)
Supplement: Multimedia Appendix 3 [file periop_v8i1e58878_app3.docx]

## Appendix C: UTAUT2 survey items

### Performance Expectancy

**PE1:** I expect using IkHerstel will contribute to my health

**PE3:** Using ikHerstel has contributed to my health

**PE4:** ikHerstel has helped me recovery the activities of my daily life

### Effort Expectancy

**EE1:** Learning how to use ikHerstel is easy for me

**EE3:** The recommendations in ikHerstel are clear

**EE4:** Entering my progress on the activities in the app is easy for me

### Facilitating conditions

**FC2:** I have the knowledge necessary to use the ikHerstel app

**FC4:** I can get help from others when I have difficulties using ikHerstel

### Habit

**HT1:** The use of ikHerstel has become a habit for me

### Price value

**PV1:** I would be willing to pay to use ikHerstel
